# Distribution of *CCR5*-*Delta32*, *CCR5 promoter 59029 A*/*G*, *CCR2*-*64I* and *SDF1*-*3*’*A* genetic polymorphisms in HIV-1 infected and uninfected patients in the West Region of Cameroon

**DOI:** 10.1186/1756-0500-6-288

**Published:** 2013-07-23

**Authors:** Céline Nguefeu Nkenfou, Linda Chapdeleine Mouafo Mekue, Christelle Tafou Nana, Jules Roger Kuiate

**Affiliations:** 1 “Chantal Biya” International Reference Centre for Research on HIV and AIDS Prevention and Management (CIRCB), P.O. Box 3077, Yaounde-Messa, Cameroon; 2 Department of Biological Sciences, Higher Teachers’ Training College, University of Yaounde I, Yaounde, Cameroon; 3 Department of Biochemistry, Faculty of Sciences, University of Dschang I, Dschang, Cameroon; 4 Department of Parasitology, Faculty of Sciences, University of Dschang I, Dschang, Cameroon

**Keywords:** HIV, AIDS related gene variants, Allelic frequency, Cameroon

## Abstract

**Background:**

Genetic variants of the genes encoding Human Immunodeficiency Virus-1 (HIV-1) co-receptors and their ligands, like CC-Chemokine Receptor 5 delta 32 mutation (*CCR5*-*Delta32*), *CCR5 promoter A*/*G* (Adenine/Guanine), CC-Chemokine Receptor 2 mutation 64 isoleucine (*CCR2*-*64I*) and the Stromal cell-derived Factor 3’A mutation (*SDF1*-*3*’*A*), are involved in the susceptibility to HIV-1 infection and progression. The prevalence of these mutations varies by Region. However, little is known about their distribution in the population of Dschang, located in the West Region of Cameroon. The prevalence of HIV in the West Region of Cameroon is lower than elsewhere in Cameroon. The objectives of this study were to determine the distribution of four AIDS Related Gene (ARG) variants in HIV-infected and non-infected population of Cameroon especially in the West Region and to estimate the contribution of these variants to the susceptibility or resistance to HIV infection. We also aimed to evaluate the effectiveness of genotyping using dried blood spot (DBS) samples.

**Methods:**

A total of 179 participants were recruited from two hospitals in Dschang in the West Region of Cameroon. Their genotypes for *CCR5*-*Delta32*, *CCR5 promoter 59029A*/*G*, *CCR2*-*64I* and *SDF1*-*3*’*A* were analyzed using polymerase chain reaction (PCR) and restriction fragment length polymorphisms.

**Results:**

A total of 179 participants were enrolled in the study. Among them, 32 (17.9%) were HIV positive and 147 (82.1%) were HIV negative. The allelic frequencies of these genes were: 0%, 49.72%, 17.6% and 100% respectively for *CCR5*-*Delta32*, *CCR5 promoter 59029A*/*G*, *CCR2*-*64I* and *SDF1*-*3*’*A*. No individual was found to carry the *CCR5*-*Delta 32* mutation. All participants recruited were heterozygous for the *SDF1*-*3*’*A* allele.

**Conclusion:**

Our data suggest that the *CCR5*-*Delta32* cannot account for the protection as it was completely absent in our population. *SDF1*-*3*’*A* variants, may be in association with other polymorphisms, may account for the overall protection from HIV-1 infection in participants recruited as everyone carries this allele. The *CCR5 promoter 59029 G*/*G* genotype may be associated with the risk for HIV-1 infection in this population, while the *CCR2*-*64I* (A/A genotype) may account for the protection against HIV infection. The results of genotyping from fresh blood and DBS were comparable.

## Background

Thirty years after the discovery of HIV as the cause of AIDS there is still no effective vaccine and no cure for this disease. HIV susceptibility shows a consistent level of individual heterogeneity, much of which can be conferred by host genetic variation, some to the virus and some to the immune response. In an effort to discover host factors required for HIV replication, to identify crucial pathogenic pathways, and to reveal the full armament of host defenses, there has been a shift from candidate-gene studies to unbiased genome-wide genetic and functional studies. However, the number of securely identified host factors involved in HIV disease remains small, explaining only approximately 15-20% of the observed heterogeneity [1].

Humans are constantly in contact with infectious pathogens. Selective pressure may result in genetic changes that may be important to escape infections or to better fight against infection [2].

The unexpected encounter, between HIV and the chemokine system has dramatically advanced our understanding of the pathogenesis of AIDS, opening new perspectives for the development of effective prophylactic and therapeutic measures [3].

A previous survey conducted in 2000 in Cameroon indicated substantial variation in HIV seroprevalence, ranging from 0% to 18% among ethnic groups in different villages of Cameroon [4]. A recent report from the national HIV/AIDS committee showed differential HIV prevalence according to the Region; the West Region where the Bamileke people live has one of the lowest rates of HIV prevalence at 2.3% [5]. Whether genetic variation among ethnic groups can account for or reflect important portions of the differences in HIV seroprevalence among groups in Cameroon is unknown. However, limited information is available about the distribution among overall African populations of host genetic polymorphisms conferring resistance to HIV-1 infection or slowing HIV disease progression. Identifying *CCR2*-*64I*, *CCR5*-*Δ32*, *CCR5 promoter 59029 A*/*G* and *SDF1*-*3*′*A* allelic variants and their distribution may help understand the burden and course of the disease which may be important in clinical decision making. In the present study, we investigated four ARG variants, in the West Region of Cameroon and analyzed the association of these host gene polymorphisms with HIV serostatus.

Notably, polymorphisms of the genes for *CCR5*, *CCR2* and stromal-derived factor 1 (*SDF1*) have been found to modulate the susceptibility of individuals to HIV-1 infection and/or the pathogenic progression [6]. However, the real effect of those gene polymorphisms on the susceptibility of individuals to HIV-1 infection and AIDS progression remains controversial.

Individuals who are homozygous for the mutations (*CCR5*-*Δ32*/*Δ32*) show high resistance to HIV-1 infection in many ethnic populations [7-10]. However, recent studies found that several individuals with the *CCR5*-*Δ32*/*Δ32* genotype were HIV-1 positive [11-13]. Furthermore, the distribution of *CCR5*-*Δ32* frequency varies in different ethnic populations. While a high frequency of *CCR5*-*Δ32* appears in Caucasians, a very low frequency is evident in Asian people, [10,14-17] and in Africa [18]. A previous report showed no *CCR5*-*Δ32* allele in seven ethnic populations in Cameroon [19].

Several other polymorphisms in the *CCR5* gene have been associated with HIV infection, namely the *CCR5*-*m303*, the *CCR5*-*59653T* and the *CCR5 promoter 59029 A*/*G*. *CCR5 promoter 59029 A*/*G* is an A/G transition identified at base pair 59029 in the *CCR5* promoter. Both promoter alleles are common (43-68% allelic frequency for 59029-A depending on race) [20]. The *CCR5 promoter 59029 A*/*G* distribution and its impact in HIV-1 infection in Cameroon have not yet been explored.

In HIV-1/AIDS, the mutation of valine (V) to isoleucine (I) in *CCR2* has not been shown to affect susceptibility to infection, but HIV-infected persons, heterozygous or homozygous for this mutation appeared to progress to AIDS or death more slowly [21]. This may not be by its action directly but, by the linkage to other haplotype mutations (example *CCR5*-∆*32*, *CCR5*-*59653T*). Some previous studies, however, have not confirmed this effect on progression to disease [22]. The delay of disease is achieved through a long asymptomatic phase of the disease. Studies on infected commercial sex workers in Nairobi, Kenya, suggested that the presence of the mutation helped to explain the slow progression in 21% to 46% of slow progressors [23]. One mutation linked to the CC-chemokine receptor, *CCR2*-*64I* is present mostly in Africa compared to the rest of world. However, despite the high prevalence of HIV in Africa, the *CCR2*-*64I* mutation alone is but one possible factor in HIV/AIDS development. The frequency of this mutation is as follows: in overall Africa (17.2%), Gambia (4.3%), and Central Africa (20.2%) [24]. In some Spanish populations the prevalence is also high (14% - 30%) [25].

SDF1 is the principal ligand of CXCR4. In *SDF1*-*3*′*A*, a G to A mutation at position 801 relative to the ATG start codon in the non-coding Region of the *SDF*-*1* gene, has been shown to inhibit AIDS progression [26,27]. HIV infected patients homozygous for this mutation exhibited a significantly delayed progression to AIDS and an even more significant decrease in mortality [27]. It has been known that the *SDF1*-*3*′*A* mutation could result in increased SDF1 production, resulting in delayed infection due to the strong competition with the CXCR-4 chemokine receptor. Other groups, however, did not observe a similar inhibitory effect, but instead reported that patients who were homozygous for the *SDF1*-*3*′*A* variant had more-rapid disease progression [28-31]. Importantly, the frequency of the *SDF1*-*3*′*A* mutation varies in different ethnic populations [32].

We are reporting in this article the distribution of four ARG’s in Dschang in the West Region of Cameroon.

## Methods

### Subjects and sample collection

Participants of both sexes were recruited from patients consulting at the district hospital and the Saint Vincent de Paul hospital in Dschang city. Only consenting participants were enrolled and a questionnaire was administered. Experimental protocols were approved by the National ethics committee under the N°269/CNE/SE/2011. Written informed consent was obtained from each participant. Five ml of blood were collected in EDTA tubes from each participant. The plasma was used for the diagnosis of HIV and the Buffy coat was used as a source of genomic DNA for genotyping.

### HIV testing

The presence of HIV antibodies was detected using Determine HIV 1/2 test (Alere, 357 Matsuhidai, Matsuda-shi, Chiba, 270–2214 Japan) and confirmed using the Genie III HIV-1/HIV-2 test (Biorad 3, Bd Raymond Poincaré, 92430 Marnes La Coquette, France).

### Isolation of genomic DNA and genotyping of *CCR5*-*Δ32*, *CCR5 promoter 59029 A*/*G*, *CCR2*-*64I* and *SDF1*-*3*′*A*

Genomic DNA was extracted from the Buffy coat using the QiaAmp DNA mini kit (Qiagen S.A 3 Avenue du Canada, LP 809, 91974 Courtaboeuf Cedex, France), according to the manufacturer’s instructions.

The *CCR5*-*Δ32*, *CCR5 promoter 59029 A*/*G*, *CCR2*-*64I*, and *SDF1*-*3*′*A* genetic variants in individual subjects were characterized by PCR followed by RFLP detection using the specific primers and restriction endonucleases as described previously [33,34]. The sequences of primers and the restriction enzymes used are presented in Table 1. All restriction enzymes used in this work were purchased from New England Biolabs, 240 County Road, Ipswich, MA, 01938–2723, USA, and used according to their instructions.

**Table 1:** **Primers**, **amplicon size**, **enzymes**, **expected fragments size after digestion and references used for genotyping**

**Genes**	**Primers sequence (5’-3’)**	**Size of the amplicon (bp)**	**Enzyme used for the RFLP**	**Expected fragments size (bp)**	**Reference**
*CCR5*-*Δ32*	CTTCATCATCCTCCTGACAATCG	262 (wt)	None	262 or 230	[33]Kristiansen *et al*., 2001
	GACCAGCCCCAAGTTGACTATC	230 (mut)			
*CCR5 promoter A*/*G*	TGGGGTGGGATAGGGGATAC	498	*Bsp*1286I	453 + 45	[33]Kristiansen *et al*., 2001
	TGTATTGAAGGCGAAAAGAATCAG				
*CCR2*-*64I*	GGATTGAACAAGGACGCATTTCCCC	380	*Fok*I	215 +165	[34]Magierowska *et al*., 1999
	TTGCACATTGCATTCCCAAAGACCC				
*SDF1*-*3*’*A*	CAGTCAACCTGGGCAAAGCC	302	*Msp*I	202+ 100	[33]Kristiansen *et al*., 2001
	AGCTTTGGTCCTGAGAGTCC				

The *CCR5*-*Δ32* gene fragment amplified by the above mentioned primers will produce two types of fragments and three types of genotypes depending on the presence or not of the mutation: a 262 bp fragment if the person does not have the Δ32 mutation on its two alleles (wild type), a 230 fragment if the person is homozygous for the deletion (mutant) and two fragments 262 and 230 if the person is heterozygous.

The *CCR5*-*promoter 59029 A*/*G* fragment amplified by the above mentioned primers is 498 bp. The A to G mutation introduces a recognition site for the *Bsp*1286I restriction enzyme. If there is mutation, then *Bsp*1286I will cleave and produce two fragments of 453 and 45 bp respectively if the person is homozygous for the mutation. If the person is heterozygous there will be three fragments on the gel of 498 bp, 453 bp and 45 bp respectively. If a person has the wild type allele, then only one fragment of 498 bp will be seen on the gel.

The *CCR2* gene fragment amplified by the primers in Table 1 is 380 bp. The 64I mutation introduces a recognition site for *Fok*I. In this case, *Fok*I will cleave at its recognition site and produce two fragments of 215 and 165 bp respectively if the person is homozygous for the mutation. If a person is heterozygous there will be three fragments on the gel at 380 bp, 215 bp and 165 bp respectively. There will be just one fragment at 380 bp if the *CCR2* gene fragment does not have the mutation.

The *SDF1*-*3*′*A* gene fragment amplified by the designed primers (in Table 1) is 302 bp. The A to G mutation removes the recognition sequence specific for *Msp*I. If there is a mutation, then the enzyme *Msp*I will not cleave and only the 302 bp fragment will be present. If the person does not carry the mutation (homozygous wild type); the *Msp*I digestion will produce two fragments of 202 and 100 bp respectively. If the person is heterozygous there will be three fragments on the gel at 302 bp, 202 bp and 100 bp respectively.

### Genotyping using DBS samples

To evaluate the performance of genotyping using DBS samples, 50 random blood samples were spotted on Whatman paper and allowed to dry at room temperature in the dark [35]. DNA extraction was done on these DBS using a QiaAmp DNA minikit according to the manufacturer’s instruction. Eluted DNA was amplified and digested as described above. This evaluation was done on the *CCR2*-*64I* gene. Results obtained were compared to those obtained from fresh Buffy coat.

### Statistical analysis

The allelic frequencies were calculated as (*h* + 2*H*)/2*N*, where *H* is the number of samples with a homozygous mutation genotype, *h* is the number with a heterozygous mutation genotype and *N* the total number of samples. The frequency was further analyzed by Hardy–Weinberg equilibrium (HWE) analysis. The HWE was performed in the population using PopGen software. The differences in the frequency of each genetic variant between HIV-1 seronegative and HIV-1 seropositive groups were determined by a Chi-square test. A value of *P* < 0.05 was considered statistically significant.

## Results

### Study population

To determine the distribution of *CCR5*-*Δ32*, *CCR5 promoter 59029 A*/*G*, *CCR2*-*64I* and *SDF1*-*3*′*A* genetic variants, we recruited 179 subjects living in Dschang in the West Region of Cameroon, who were either HIV-1 seropositive or seronegative. They were all aged between 16 and 81 years old, with the median age of 27. There were 123 (68.7%) female and 56 (31.3%) male. The epidemiological characteristics of the subjects are presented in Table 2.

**Table 2:** **Demographic characteristics of study participants according to the HIV**-**1 sero status**, **age and sex**

	**Female**		**Male**		**Total**		**Overall**
**Age group**^**a**^	**HIV+**	**HIV-**	**HIV+**	**HIV-**	**Female**	**Male**	
16-24	6	43	3	21	49	24	73
25-34	10	36	0	17	46	17	62
35-44	8	13	4	2	21	6	27
45-54	1	2	0	9	3	9	12
55-81	0	4	0	0	4	0	5
Total	25	98	7	44	123	56	179

a.years.

### HIV serology

From the 179 participants, 147 (82.1%) were HIV negative. Thirty two were confirmed to be HIV positive (17.9%) of which 25 were female and 7 were male. 41% of the HIV positive participants were already on ARV treatment.

### Evaluation of the genotyping using DBS

DNA extraction was done successfully from dried blood spots. The amplification of the 380 bp of the *CCR2* gene fragment was done according to the method developed by Magierowska [34]. The results obtained were 100% concordant with those obtained from fresh blood. So DBS samples can be confidently used for genotyping.

### Genotyping

The allelic frequencies of *CCR5*-*Δ32* (0%), *CCR5 promoter 56029 A*/*G* (49.72%), *CCR2*-*64I* (17.60%) and *SDF1*-*3*′*A* (100%) in the population were reported. These results are presented in Table 3 for the whole population and Table 4 according to the HIV serostatus.

**Table 3:** **Distribution of *CCR5***-***Δ32***, ***CCR5 promoter 59029 A***/***G***, ***CCR2***-***64I *and *SDF1***-***3***′***A *in HIV seropositive and seronegative groups**

**Gene variants**		**HIV positive N (%)**	**HIV negative N (%)**
*CCR5*-*Δ32*	Wt/wt^a^	32 (100)	147 (100)
	Wt/Δ32^b^	0 (0.0)	0 (0.0)
	Δ32/Δ32^c^	0 (0.0)	0 (0.0)
*CCR5 promoter 59029 A*/*G*	A/A^a^	5 (15.62)	29 (19.72)
	A/G^b^	17 (53.12)	95 (64.62)
	G/G^c^	10 (31.25)	23 (15.64)
*CCR2*-*64I*	G/G^a^	24 (75)	97 (66.1)
	G/A^b^	8 (25)	45 (30.6)
	A/A^c^	0 (0.0)	5 (3.4)
*SDF1*-*3*’*A*	G/G^a^	0 (0.0)	0 (0.0)
	G/A^b^	32 (100)	147 (100)
	A/A^c^	0 (0.0)	0 (0.0)

a. Wild type homozygotes.

b Heterozygotes.

c Mutant type homozygotes.

**Table 4:** Allelelic frequencies of the four ARG^*^variants in the study population

**Alleles**	**Quantity (n)**	**Subjects (N)**	**Allelic frequency (p)**
*CCR5*- *Δ32*	0	179	0
*CCR5promoter 59029 wt*^*a*^	180	146	0,503
*CCR5promoter 59029 mt*^*b*^	178	145	0,497
*CCR2 wt*^*a*^	295	174	0,824
*CCR2 mt*^*b*^	63	58	0,176
*SDF1*-*3*’*A*	179	179	1

^*^ AIDS related gene. a. Wild type. b Mutant.

No *CCR5*-*Δ32* mutation was identified in our study group either as homozygous or heterozygous. For the *CCR5 promoter 59029 A*/*G*, prevalence of 62.6% of heterozygous, 17% of wild type and 18.4% double mutant were identified in our study group. This is the first description of the prevalence of this ARG variant in Cameroon. The prevalence of 2.7%, 29.6% and 67.6% was found respectively for the *CCR2*-*64I* homozygous mutant, heterozygous and homozygous wild type. For the *SDF1*-*3*′*A* gene neither wild type nor homozygous mutants were seen. Instead all 179 participants exhibited a heterozygous genotype.

The HWE analysis revealed that all genetic variants for *CCR5 promoter 59029 A*/*G and CCR2*-*64I* in the studied population were in equilibrium (*P* > 0.05) (Table 5).

**Table 5:** **Allele frequency and HWE**^***a ***^**analysis of four ARG**^***b ***^**in a population of West Cameroon**

**Gene variants**		**HIV positive**			**HIV Negative**			**Total Freq (%)**
	genotype	Freq (%)	*X*^2^	P	Freq (%)	*X*^2^	P	
*CCR5*-*Δ32*	Wt	100			100			100
	Δ32	0			0			0
*CCR5 promoter A*/*G*	A	42.2	0.54	0.675	51.4	12.6	0.001	46.8
	G	57.8			48.6			53.2
*CCR2*- *64I*	G	87.5	0.74	0.207	81.3	0.16	0.75	84.4
	A	12.5			18.7			15.6
*SDF1*-*3*’*A*	G	50	300.25		50	300.25		50
	A	50			50			50

a. Hardy-Weinberg equilibrium. b AIDS related gene.

### Allelic frequencies and HIV serostatus

Allelic frequencies in relation to the HIV serostatus are presented in Table 6. The allele frequency of the *CCR5 promoter 59029 A*/*G* in HIV-1 seropositive group (57.8%) was not significantly different from that (48.6%) in the seronegative group (*P* = 0.19). Importantly, there was a significant difference in G/G genotype between HIV-seropositive and HIV seronegative (34.3% and 15.6% respectively, P= 0.049), suggesting that the *CCR5 promoter 59029 G*/*G* allele may be associated with the risk of HIV-1 infection in our population. This is contradictory to a previous study where the *CCR5 promoter 59029*-*G*/*G* appears to be protective relative to *CCR5 59029*-*A*/*A*[20].

**Table 6:** **Allelic frequencies of *CCR5 promoter A***/***G *and *CCR2 64I *and HIV serostatus in a population of west Cameroon**

**Allelic genotypes**		**HIV Seropositive**			**HIV Seronegative**			**Total**
		Freq^a^%	*x*2	P	Freq%	*x*2	P	Freq^a^%
CCR5 P* *A*/*G*	*CCR5 wt*	42,19	0,747	0,387	52,04	0,044	0,832	50,27
	*CCR5 mt*	57,81	0,794	0,372	47,96	0,034	0,853	49,72
*CCR2 64I*	*CCR2 wt*	87,5	0,834	0,36	81,3	0,979	0,322	82,38
	*CCR2 mt*	12,5	0,476	0,49	18,7	0,662	0,415	17,62

*. Promoter.

a. Frequency.

The allele frequency of *CCR2*-*64I* in the HIV-1 seronegative group (18.7%) was not significantly higher than that (12.5%) in seropositive group (*P* = 0.22). This suggests that the *CCR2*-*64I* allele is unlikely to be associated with the low prevalence of HIV-1 infection in our population.

## Discussion

In our study we have recruited a total of 179 participants, on which the HIV serology test has been performed. We obtained 32 HIV positive patients and 147 HIV negative ones. This gave a seroprevalence of 17.9% higher than the general prevalence of the west Region that is at 2.3% [5]. This is explained by the fact that our participants were enrolled among patients consulting at hospitals.

The evaluation of the methodology using DBS in comparison to fresh blood sample was done using the test of Kappa concordance. The kappa of concordance was 0.95 (p = 0.00), which means that there was a high concordance between the two methods. So DBS can be used for ARG genotyping.

Studies have revealed a differential distribution of ARG variants according to ethnicity [32]. In particular, it was reported that the frequency of the *CCR5*-*Δ32* was high in Caucasians, but low or even absent in Asians and Africans [18]. The distribution of the *CCR2*-*64I* allele in South Africans was 13%, and in African populations of Kenya it ranged from 21% to 23%, [23,36] and in 7 ethnicities of Cameroon from 0 to 7.1% [19]. The frequency of *SDF1*-*3*′*A* ranged widely across ethnic groups, from 3% to 71% worldwide and from 3% to 9% in Africans [32]. Data on the different genetic frequencies of *CCR5*-*Δ32*, *CCR5 promoter 59029 A*/*G*, *CCR2*-*64I* and *SDF1*-*3*′*A* alleles reported in African populations living in different African countries are however sparse. The present study is the second to describe the distribution of *CCR5*-*Δ32*, *CCR2*-*64I* and *SDF1*-*3*′*A* in Cameroon. It is the first to describe these distributions in the West Region and that of the *CCR5 promoter 59029 A*/*G* in Cameroon.

Since no *CCR5*-*Δ32* allele was found in our population it could not account for the low HIV prevalence in the West Region of Cameroon. As shown by the study of Ma [19], *CCR5*-*Δ32* is extremely rare in Cameroon (0% as in our study population).

About 50% (49.72%) of our population carry the *CCR5 promoter 59029 A*/*G* mutation. Looking at the distribution of *CCR5 promoter 59029 A*/*G* between the HIV positive and HIV negative, the allelic frequency was the same for the HIV positive and the HIV negative group. But the homozygote G/G seems to be higher in HIV positive group, contradicting previous findings [20].

The distribution of the *CCR2*-*64I* in our study population was 17.6%, compared to 31.3% obtained by Ma [19], from 7 ethnic populations in the South and Central Regions of Cameroon. This mutation is thus high in the Central and South Regions of Cameroon compared to the West Region.

Because in HIV positive and HIV negative individuals the distribution of this allele was the same, this mutation confers no resistance to HIV infection but may be involved in the progression of HIV infection to AIDS. Ma [19]*et al*. in 2005 found that *CCR2*-*64I* delays progression of HIV disease to AIDS and death in men and not in women, by conditional logistic regression analysis with matching on ethnicity and adjustment for differences in age. But a meta-analysis, based on all the published works on *CCR2*-*64I* in relation to susceptibility to HIV infection, has concluded that this allele has no effect on reducing the risk of HIV-1 infection [37]. Instead, it may act with *CCR5*-∆*32* as haplotypes in delaying HIV infection progression to AIDS [34,38].

We have obtained 100% frequency of *SDF1*-*3*′*A* in our study population compared to 0–7.1% obtained by Ma *et al*., *2005*. Could this high prevalence contribute either alone or linked to another gene as a haplotype to the low prevalence of HIV in this group? This result may be specific to the population studied and also to the small size of the population that may be not representative. We intend to extensively investigate the occurrence of this gene in all 10 Regions of Cameroon and on a larger population. Such a larger study may lead to an explanation for our current results. If the exclusive presence of this one allele is confirmed, this may have been selected in an early population that was exposed to HIV or some related disease and the beneficial mutation transmitted from generation to generation. A high frequency of *SDF1*-*3*′*A* (71%) was also observed in the Oceanian population in New Guinea, [32] where the human presence dates back at least 40,000 years to the oldest human migrations out of Africa. The prevalence of HIV in Papua New Guinea was 1.5% in 2007 and it decreased to 0.9% in 2009.

The *CCR5*-∆*32* and *CCR2*-*64I* alleles have been shown to have a strong protective effect on progression of HIV-1 infection, but *SDF1*-*3*′*A* homozygosity carried no such protection [31]. This mutation is associated with the onset of AIDS progression. The protective effect of *CCR2*-*64I* is dominant, whereas that of *SDF1*-*3*'*A* is recessive. Surprisingly, the *SDF1*-*3*'*A* homozygote has also shown a possible protection against HIV-1 infection [26,27]. Based on the small size population studied, *SDF1*-*3*′*A* heterozygosity may contribute to low HIV/AIDS infection in the West Region of Cameroon.

The combinations of the genotype at loci *CCR5* promoter and *CCR2* in HIV positive individuals and HIV negative individuals were analyzed. As shown in the Table 7, P values of 0.760, 0.134, 1, 0.305 and 0.814 were obtained for the five combinations. These results demonstrate that none of the combinations can account for the low prevalence of HIV in the West Region of Cameroon.

**Table 7:** **Combination of the gene variants of *CCR5 promoter A***/***G *and *CCR2 64I *and HIV serostatus in a population of west Cameroon**

CCR5 P*	***CCR2***-***64I***	**HIV Seropositive (n=32)**	**HIV Seronegative (n=147)**	**P-value**
Wt (A/A)	Wt (G/G)	4	16	0.760
Mt (G/G)	Wt (G/G)	9	24	0.134
Wt (A/A)	Mt (A/A)			
Hetero (A/G)	Mt (A/A)	1	4	1
Mt (G/G)	Hetero (G/A)			
Hetero (A/G)	Wt (G/G)	12	70	0.305
Wt (A/A)	Hetero (G/A)			
Mt (G/G)	Mt (A/A)	0	0	
Hetero (A/G)	Hetero (G/A)	6	33	0.814

*. Promoter.

Wt: wild type, Mt: mutant, Hetero: heterozygote.

The *χ*^2^ tests show that the *CCR5 promoter 59029 A*/*G* and *CCR2*-*64I* polymorphism were in equilibrium from generation to generation (the *X*^*2*^ value > 3.84). The observed genotype frequencies had no significant difference from the frequencies expected in each group of polymorphisms. In light of the prevalence of these mutations we can say that these mutations were present in our population since early times. The presence of these mutations worldwide would imply that these mutations arose prior to the dispersal of modern *Homo sapiens* from an African ancestral population more than 100 000 years ago [32].

It is known that following infection with HIV-1, the rate of clinical disease progression varies between individuals. Factors such as host susceptibility, genetics and immune function [39], health care and co-infections [40], as well as viral genetic variability [41], may affect the rate of progression to AIDS. Many studies have looked at the genetic viral distinctions that might explain delayed HIV progression. Considerable research investigating the viral genotype has focused on deletions in the *nef* gene of HIV seen in some nonprogressors. While many researchers have observed defective *nef* gene alleles in long term nonprogressors (LTNP), others have not [42-45]. Other researchers have focused on human leukocyte antigen (HLAmolecules [34,46,47]. All these factors interplay to affect the acquisition the resistance or the progression into HIV/AIDS.

## Conclusion

In summary, we investigated the distribution of four ARG variants in HIV seropositive and seronegative population. Not surprisingly, we found no *CCR5Δ32* allele. Surprisingly, all the subjects exhibited the *SDF1*-*3*′*A* mutation as heterozygous. The frequency of *CCR5 promoter G*/*G* in HIV seropositive was significantly higher than that in HIV seronegative. Therefore, these data suggest that the *CCR5 promoter G*/*G* variant together with other factors may be associated with a risk for HIV infection. The distribution of *CCR2*-*64I* was similar in HIV positive and HIV-negative group, and could not be attributed any impact on HIV in our population.

From the study done on the four ARG variants and in light of the limited size of the population, further studies are needed on a larger population to provide more conclusive results and to define the role if any of *SDF1*-*3*′*A* in HIV prevalence in our population. Other ARG should also be studied including HLA gene families, as the effects of the gene variants are likely to be associative.

In light of the disparities in the distribution of ARG by ethnicity, and the contradictory effect of these ARG’s on HIV susceptibility or progression, we suggest that studies be done on more population groups and that ethnicity must be taken into account. A more extensive meta-analysis on larger data sets may lead to conclusive results.

## Abbreviations

A: Adenine; AIDS: Acquired Immunodeficiency syndrome; ARG: AIDS Related Genes; CCR2: CC-Chemokine Receptor 2; CCR5: CC-Chemokine Receptor 5; CXCR4: CX-Chemokine Receptor 4; DBS: Dried Blood Spot; DNA: Deoxyribonucleic acid; G: Guanine; HIV: Human Immunodeficiency virus; I: Isoleucine; PCR: Polymerase Chain Reaction; RFLP: Restriction Fragment Length Polymorphism; SDF: Stromal cell-derived Factor; V: Valine.

## Competing interests

We have no conflicts of interest in the conduct of this study.

## Authors’ contributions

All authors contributed in the study and they have all approved the final version for publication. CNN: Conception, design, data analysis, manuscript preparation and revision, LCMM: Sample analysis, data acquisition, analysis and manuscript revision, CTN: Sample collection, data acquisition and manuscript revision, JRK: Critical manuscript revision. All authors read and approved the final manuscript.
